# Surgical rescue for persistent head and neck cancer after first-line treatment

**DOI:** 10.1007/s00405-020-05807-0

**Published:** 2020-01-25

**Authors:** Teresa Bernadette Steinbichler, L. Golm, D. Dejaco, D. Riedl, B. Kofler, C. Url, D. Wolfram, H. Riechelmann

**Affiliations:** 1 Department of Otorhinolaryngology-Head and Neck Surgery, Medical University of Innsbruck, Anichstr. 35, 6020 Innsbruck, Austria; 2 Department of Medical Psychology, Medical University of Innsbruck, Anichstr. 35, 6020 Innsbruck, Austria; 3 Department of Plastic, Reconstructive and Aesthetic Surgery, Medical University of Innsbruck, Anichstr. 35, 6020 Innsbruck, Austria

**Keywords:** Salvage surgery, Pectoralis major flap, VAC therapy, Clavien–Dindo score, Salvage laryngectomy

## Abstract

**Purpose:**

Surgical rescue is a treatment option for persistent disease after first-line treatment treatment of head and neck cancer (HNC).

**Methods:**

Patients with persistent HNC treated with rescue surgery between 2008 and 2016 were included. Patients who received a rescue neck dissection (ND only) and who received primary site surgery ± ND were analysed separately (primary site surgery ± ND).

**Results:**

During the observation period, 35 patients received ND only and 17 primary site surgery ± ND. No perioperative mortality was observed. In nine patients with ND only and 12 patients with primary site surgery ± ND at least one complication was encountered. 41/52 (79%) patients had a complete response. Median overall survival of patients receiving rescue surgery was 56 months (95% CI 44–69 months). Median overall survival was best for patients with initial laryngeal and oropharyngeal cancer and worst for patients with hypopharyngeal cancer (*p* = 0.02). Functional deficits following rescue surgery were mainly observed in the domains speech, nutrition, and shoulder/arm mobility. The risk of functional impairment was higher for patients with rescue surgery at the primary tumor site (OR 2.5 ± 2; *p* = 0.07).

**Conclusion:**

Rescue surgery offers patients with resectable, persistent disease a realistic chance to achieve long-term survival. Especially patients with laryngeal and oropharyngeal cancer profited from rescue surgery. Rescue neck dissection is an effective and safe procedure. Patients with rescue surgery at the primary tumor site ± ND should expect complications and permanent functional impairment.

**Electronic supplementary material:**

The online version of this article (10.1007/s00405-020-05807-0) contains supplementary material, which is available to authorized users.

## Introduction

In patients with advanced stages of head and neck carcinoma (HNC), radiochemotherapy is often preferred as first-line therapy to achieve organ preservation [1]. A prerequisite for cure is the complete response to antitumor treatment [2]. Incomplete response to first-line treatment is a common problem in HNC and occurs in 20–25% of the patients with incident HNC [1, 3, 4]. Re-irradiation is usually not an option, as radioresistant clones have been selected during first-line therapy and re-irradiation is associated with severe tissue damage especially shortly after previous high-dose therapy [5]. Chemotherapy alone is not considered a curative approach [6]. Second-line surgery, also referred to as rescue or salvage surgery, offers a realistic chance for patients with resectable persistent disease to finally achieve CR despite first-line treatment failure [3, 6]. However, surgical second-line treatment of HNC carries a high risk of mortality and morbidity.

In a previous study, we reported that patients treated with rescue surgery following first-line treatment failure had the best overall survival compared with patients treated with any other second-line treatment modality [3]. Here, we analysed the outcome of rescue surgery in detail. We were interested in the duration of surgical procedures, intensive care unit (ICU) treatment and hospital stay, perioperative mortality, postoperative complications, complete response (CR) rate and overall survival as well as long-term functional outcome after rescue surgery. Patients with only cervical lymph node persistence (ND only) and patients with persistence at the primary tumor site with or without neck involvement (primary site surgery ± ND) were analysed separately.

## Materials and methods

### First-line treatment

First-line treatment of HNC patients at the Department of Otorhinolaryngology-Head and Neck Surgery, Medical University of Innsbruck, Austria, was recommended by an interdisciplinary tumor board (ITB) in line with National Comprehensive Cancer Network (NCCN) Guidelines [6]. All patients were routinely assessed for treatment response 8–12 weeks after the end of first-line treatment [3, 7]. Treatment response evaluation included contrast enhanced CT, MRI or PET-CT scans and a restaging endoscopy usually under general anaesthesia with biopsies from the initial tumor sites [8]. Results of treatment response evaluation were presented in the ITB. In case of persistent HNC, second-line therapies or BSC were advised. In line with recent NCCN guidelines, rescue surgery was favored if tumors were considered resectable [6]. Local residual tumor resection was frequently combined with free flap reconstruction. Neck dissections were either performed during resection of the residual primary tumor or as a sole treatment, if persistent disease was limited to the neck [9].

### Inclusion and exclusion

All patients with HNC treated between 2008 and 2016 were screened for inclusion. Patients with persistent HNC after first-line therapy who received rescue surgery between 01 January 2008 and 31 December 2016 were included. Patients with recurrent disease following a previous complete response (CR) were excluded. Only patients with HNC, except carcinomas of the thyroid gland, oesophagus, eye, brain, spine or skin, were eligible. Patients with distant tumors metastasizing to the head and neck region were also excluded.

### Clavien–Dindo classification

Severity of complications after rescue surgery was classified according to the Clavien–Dindo classification [10]. The Clavien–Dindo classification ranks surgical complications in an objective and reproducible manner [10]. We did not use the subclasses ‘a’ and ‘b’ and used an abbreviated five-grade classification due to a limited number of patients in the study cohort [10, 11]. If a patient had more than one complication with different Clavien–Dindo grades, the complication with the highest Clavien–Dindo grade was counted (Table 4).

### Grouping and prognostic factors

Included patients were grouped into patients with cervical lymph node persistence only treated with rescue neck dissection (ND only) and patients with persistence at the primary tumor site with or without neck involvement (primary site surgery ± ND). The extent of surgical procedures at the primary tumor site was further subdivided into transoral/transnasal surgery ± ND, laryngectomies ± ND routinely performed with a myofascial pectoralis major flap and external approaches of the primary tumor with free tissue transfer (all with ND). Moreover, various disease parameters were recorded and categorized including patient age and sex, American Society of Anaesthesiologist Physical Status (ASA) score as a simple measure of comorbidity [12], initial tumor site and stage according to UICC classification version 7 [13] and first-line treatment. Immunohistochemistry was used to assess the p16 status [14]. It was defined positive when more than 60% of the cancer cells were stained positive; otherwise p16 was categorized as negative [15].

### Head and neck cancer functional integrity scale (HNC-FIT scale)

In 2016, the Functional Integrity of Head and Neck Cancer Scale (HNC-FIT Scale) was introduced at our institution. It is completed by the physician at regular follow-up visits and mainly serves for internal quality control. The HNC-FIT Scale covers the functional domains nutrition, respiration, speech, pain, mood, and neck and shoulder mobility. The functional domains are categorised into five integrity grades ordered by the extent of functional integrity, with higher grades indicating a higher degree of functional integrity. The HNC-FIT Scale questionnaire was completed at each oncological follow up visit. It is detailed in Suppl. 1. The last available HNC-FIT Scale outcome was used for functional outcome analysis. The results of the HNC-FIT survey instrument were then dichotomized. Scores 3 and 4 (slightly restricted and normal) were assigned to one group, scores 0, 1 and 2 (worst, very poor, poor) to the other group. The number of patients in these two groups was tabulated. These frequencies were then compared for each functional domain with the Fisher exact test. For graphical presentation, the percentage of patients with slightly restricted or normal functions at the last follow-up was depicted as a line graph. The obtained profile line indicates the percentage of patients who achieved complete or almost complete functional integrity, broken down by functional dimension.

### Data analysis

Median follow up time was calculated using the inverse Kaplan–Meier method [16]. Frequencies were tabulated and compared with Chi-square or Fisher’s exact tests. For metric data, medians and 25th to 75th percentiles are provided unless stated otherwise. Differences in medians were compared with Wilcoxon–Mann–Whitney tests. Overall survival was analysed with the Kaplan Meier method and compared with log rank tests. Statistical analyses were performed using SPSS 24 (IBM Corporation, Armonk, NY, USA).

## Results

### Characteristics of patients treated with rescue surgery

From January 2008 to December 2016, 52 patients with persistent disease after first-line therapy were treated with surgical rescue (Table 1). Of the 52 patients 43 were male (83%). The mean (± SD) age was 60 (± 10) years. First-line treatment had consisted of radiotherapy ± systemic therapy in 41/52 (79%), surgery and postoperative radiotherapy ± systemic therapy in 9/52 (17%) and surgery in 2/52 (4%). Ten of the patients receiving rescue surgery had a p16-positive tumor. A ND only was performed in 35/52 patients, primary site surgery ± ND was performed in 17/52 patients. Neck dissection was performed unilaterally in 39/52 (75%) and bilaterally in 6/52 (11%) patients. Surgical rescue at the primary tumor site included laryngectomies (6/52), which were routinely performed with a myofascial pectoralis major flap (5/6), transoral (4/52) or transnasal (1/52) resection of local persistent tumor or an open resection of the persistence at the primary tumor site (6/52) usually combined with free tissue transfer (5/6; Table 2). Median follow up was 55 months (95% CI 45–65 months).

**Table 1:** Characteristics of 52 patients treated with persistent disease after first-line therapy who were treated with surgical rescue

Variable	Value	Count	Percent (%)
Sex	Male	43	83
	Female	9	17
Age at diagnosis	< = 50	9	17
	51–60	17	32
	61–70	19	36
	71–80	6	11
	> 80	1	2
ASA I/II vs. ASA III/IV	ASA I/II	18	35
	ASA III/IV	22	42
Initial tumor site	Lip/oral cavity	8	15
	Oropharynx	20	39
	Hypopharynx	6	12
	Larynx	10	19
	Other^a^	8	15
Initial clinical stage	Stage II	4	8
	Stage III	8	15
	Stage IVa	33	64
	Stage IVb	6	12
	Stage IVc^b^	1	2
Site of persistence	Primary site	12	23
	Neck only	18	35
	Primary site and neck	7	13
	Distant ± any site	15	29
p16-IHC	Negative	32	76
	Positive	10	24
First-line treatment	Surgery only	2	4
	Surgery and postoperative RT	3	6
	Surgery and systemic therapy/RT	6	12
	Systemic therapy/RT	36	69
	Radiotherapy	4	8
	Radioimmunotherapy	1	2

After first-line therapy, 175/741 (24%) patients had persistent HNC. From these patients 52 were eligible for rescue surgery according to the decision of the interdisciplinary head and neck tumor board (52/175) [3]

^a^One patient with carcinoma of the paranasal sinus, seven patients with cervical cancer of unknown primary (CUP)-syndrome

^b^Patient with persistent laryngeal carcinoma and solitary pulmonary metastasis who received rescue laryngectomy and additional stereotatic body radiotherapy of the lung, had a complete response after rescue treatment

**Table 2:** Overview of surgical rescue procedures

Surgical procedure	Without neck dissection	With neck dissection	Total
Transoral/transnasal surgery	3	2	5
Laryngectomy^a^	2	4	6
External approach with tissue transfer	2	4	6
Neck dissection only	N.a	35	35

^a^Five patients with myofascial pectoralis major flap

### Total surgical procedure time

**Fig. 1:**
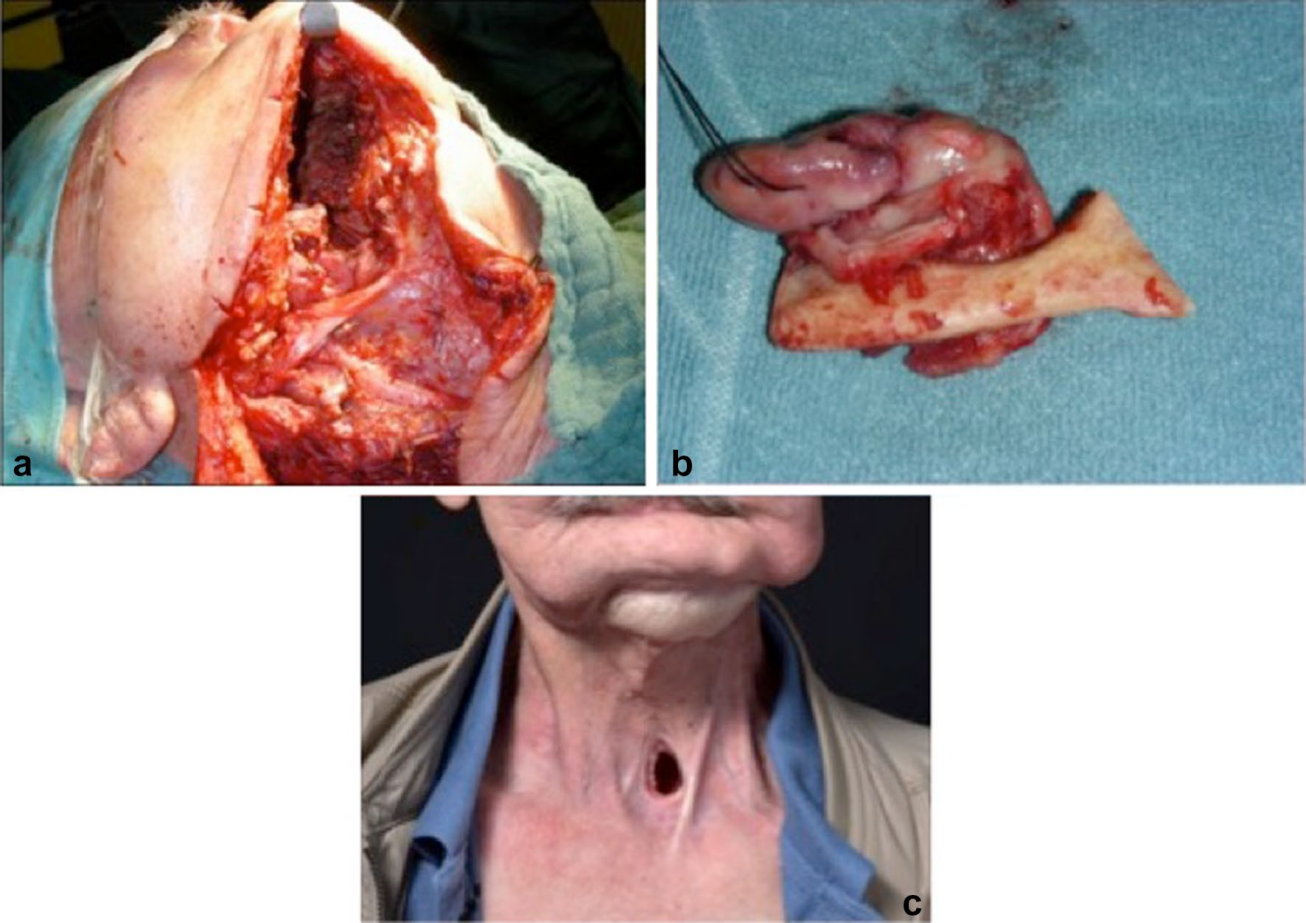
Rescue surgery in a patient with a carcinoma of the oral cavity. Patient with tumor persistence of a flour of mouth carcinoma after radiochemotherapy treated with rescue surgery. **a** Intraoperative situs during resection of a carcinoma of the floor of the mouth, marginal mandibular resection, ipisilateral modified radical neck dissection, tracheostomy and reconstruction with a latissimus dorsi flap. **b** surgical specimen **c** picture of the patient in the long-term follow up. To date the patient is still alive

The median total surgical procedure time was 182 (136 to 280; Table 3) minutes [17]. The median duration differed significantly between ND only (177; 125–206 min) and primary site surgery ± ND (290, 174–613 min; *p* < 0.001; Table 3). Total surgical procedure time did not correlate with the initial tumor site (*p* = 0.3). The median duration of the surgical procedure was significantly longer if tissue transfer was performed (609, 75th percentile 880 min; *p* < 0.001). Ten patients received a reconstruction with free tissue transfer (5/10) or a pedicled myofascial pectoralis major flap (5/10) at the primary tumor site. The most common used free flap was a radial forearm flap (2/10) or an anterolateral thigh flap (2/10). One patient received a free latissimus dorsi flap (1/10; Fig. 1). One patient with ND only received a split skin graft from the thigh due to cutaneous infiltration of cervical lymph node metastasis.

**Table 3:** Total surgical time, number of days on an intensive care unit and total number of inpatients days depending on the type of rescue surgeries

Type of primary site surgery	Count	Total surgical procedure time in minutes^a^	Days on intensive care unit	Total number of inpatient days^a^	Patients with complications
ND only	35	177 (125–206)	N.a.^b^	9 (6–13)	9
Laryngectomy^c^	6	400 (276–574)	2 (1–5)	46 (36–85)	5
External approach with tissue transfer	6	731 (526–1017)	1 (1–3)	49 (40–61)	5
Transnasal/transoral surgery	5	133 (62–257)	N.a.^b^	8 (6–24)	2

^a^Median (25. to 75. percentile)

^b^ND only: 7/35; (20%) received one day of postoperative intensive care unit treatment transnasal/transoral surgery: 1/6 (17%) received one day of postoperative intensive care unit treatment

^c^Five patients with myofascial pectoralis major flap

### Intensive care unit treatment and duration of the whole inpatient course

Thirty-eight percent of the patients received ICU treatment postoperatively (20/52; Table 3) Seven patients with ND received ICU treatment postoperatively (7/35; 20%). Twelve patients (12/17, 70%) with primary site surgery ± ND spent median 1 (0.5–2.5) day on an ICU. The probability of postoperative ICU treatment was three times higher if patients received rescue surgery at the primary tumor site instead of rescue ND only (OR 3.1 ± 0.46, *p* = 0.01). Postoperative ICU treatment correlated significantly with the duration of the surgical procedure as well as with the requirement of tissue transfer (*p* < 0.001) but it did not correlate with the initial tumor site (*p* = 0.8).

The median length of the total hospital stay was 11 (7–22) days. Twelve patients (12/52, 23%) received more than one hospital admission due to complications after rescue surgery. Seven of them had a rescue surgery at the primary tumor site (7/12, 58%). Patients who received ND only stayed median 9 (6–13) days; patients who received primary site surgery ± ND stayed median 40 (12–52) days (*p* = 0.01; Table 3). The whole inpatient stay was significantly longer if the surgical procedure was longer (*p* < 0.001), the patient had higher UICC stages at initial diagnosis (*p* < 0.05), the tumor was p16 negative (*p* = 0.05) and if a flap reconstruction with a free flap or a pedicled flap was performed (*p* < 0.05). Sex, initial tumor site and ASA score did not influence the duration of the hospital stay.

### Mortality and morbidity of surgical procedures

No perioperative mortality was observed. Twenty-one patients had at least one complication after rescue surgery (21/52, 40%). The severity of complications was graded with the Clavien–Dindo classification [18]. If patients suffered from several complications, only the complication with the highest Clavien–Dindo score was counted. Slight deviations from the normal course that required no intervention or pharmacological intervention (Clavien–Dindo grade I and II) occurred in 7/52 (13%) patients. Grade III complications requiring invasive intervention were observed in 10/52 (19%) patients, grade IV, i.e. life-threatening complications were encountered in 4/52 (8%) patients (Table 4).

**Table 4:** Clavien–Dindo classification of postoperative complications [10]

Grade	Definition	Patients with complications ND only	Patients with complications primary site surgery ± ND	Total (percent)
I	Any deviation from the normal postoperative course without the need for pharmacological treatment or surgical, endoscopic or radiologic interventions Allowed therapeutic regiments are: antiemetics, antipyrectics, analgetics, diuretics, electrolytes, and physiotherapy. This grade also includes wound infections opened at the bedside	3/35 (9%)	1/17 (6%)	4/52 (8%)
II	Requiring pharmacological treatment with drugs other than such allowed for grade I complications. Blood transfusions and total parenteral nutrition are also included	1/35 (3%)	2/17 (12%)	3/52 (6%)
III	Requiring surgical, endoscopic or radiological intervention	4/35 (11%)	6/17 (35%)	10/52 (19%)
IV	Life-threatening complication (including central nervous system complications requiring ICU management)	1/35 (3%)	3/17 (18%)	4/52 (8%)
V	Death of the patient	–	–	-

The Clavien–Dindo grade correlated with the prior received RT dose in Gray (*p* = 0.05) and with the extent of rescue surgery (*p* = 0.1). Patients with rescue surgery at the primary tumor site ± ND were 3.5 times more likely to have postoperative complications than patients with ND only (OR 3.5 ± 0. 8; *p* = 0.01). The occurrence of complications did not correlate with the ASA score (*p* = 0.6) and sex (*p* = 0.3) of the patient, age of the patient (*p* = 0.2) and p16 status of the patient (*p* = 0.15), but it correlated significantly with the initial UICC stage (*p* < 0.05).

Patients with initial laryngeal cancer had a lower Clavien–Dindo grade than patients with any other initial tumor site, but this was not statistically significant (*p* = 0.1).

The most common complication was impaired wound healing (9/52). It occurred in 2/35 patients with ND only and in 7/17 patients with primary tumor resection ± ND. The second most common complication was a secondary haemorrhage in 7/52 patients. Four of these patients had ND only, three had primary tumor site surgery ± ND. Two patients developed postoperative pneumonia (2/52), one patient suffered from a pharyngocutaneous fistula after rescue laryngectomy (1/52), one patient from flap necrosis (1/52) and one patient from postoperative thrombosis (1/52). Three patients (3/52, 6%) were not discharged home, but were transferred to a community hospital or rehabilitation centre for further roboration.

### Impact of surgical rescue on overall survival and survival following rescue surgery

Following rescue surgery 41/51 patients had a CR (79%). Patients without CR, i.e. close or involved resection margins after rescue surgery were submitted to systemic therapy (10/11) and one patient with close margins received brachytherapy of a floor of mouth carcinoma after rescue surgery (1/11). Median overall survival of patients receiving surgical rescue therapy was 56 months (44–69 months). Median overall survival was best for patients with initial laryngeal cancer (72; 46–99 months) and patients with oropharyngeal cancer (63; 46–80 months). Patients with hypopharyngeal cancer had a median overall survival of 21 months (15–26 months) and patients with cancer of the oral cavity had a median overall survival of 28 months (16–40 months; *p* = 0.02).

**Fig. 2:**
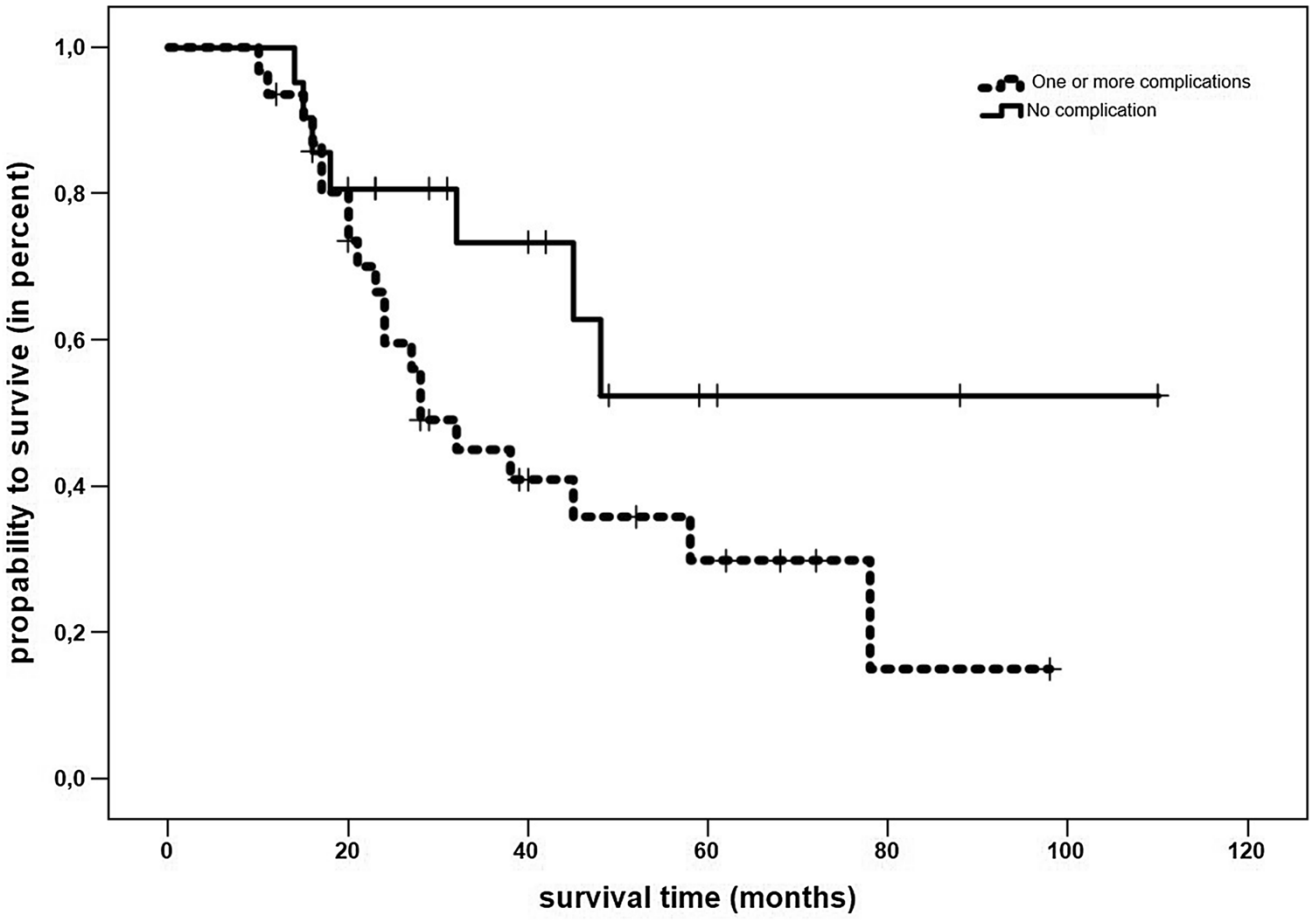
Complications and overall survival. Kaplan–Meier plot comparing overall survival in 52 head and neck cancer patients treated with rescue surgery grouped by the occurrence of complications. Patients with no complications had a better overall survival of 73 months (95% CI 51–94 months) than patients with one or more postoperative complications according to the Clavien Dindo classification (45 months, 95% CI 33–57 months, Log Rank *p* = 0.05)

No difference in overall survival was observed between male and female patients (*p* = 0.7), and patients who received ND only and patients with primary tumor site surgery ± ND (*p* = 0.5). Median overall survival after rescue surgery was significantly better for patients with an ASA score I/II (69 months, 95% CI 50–86 months) than for patients with an ASA score III/IV (42 months, 95% CI 29–55 months, *p* = 0.04). Patients who had no complication after rescue surgery had a better median overall survival of 73 months (95% CI 51–94 months) than patients suffering from one or more complications (45 months, 95% CI 33–57 months; *p* = 0.05; Fig. 2). P16 status had no significant impact on overall survival after rescue surgery (*p* = 0.3).

### Impact of surgical rescue on functional outcome

**Fig. 3:**
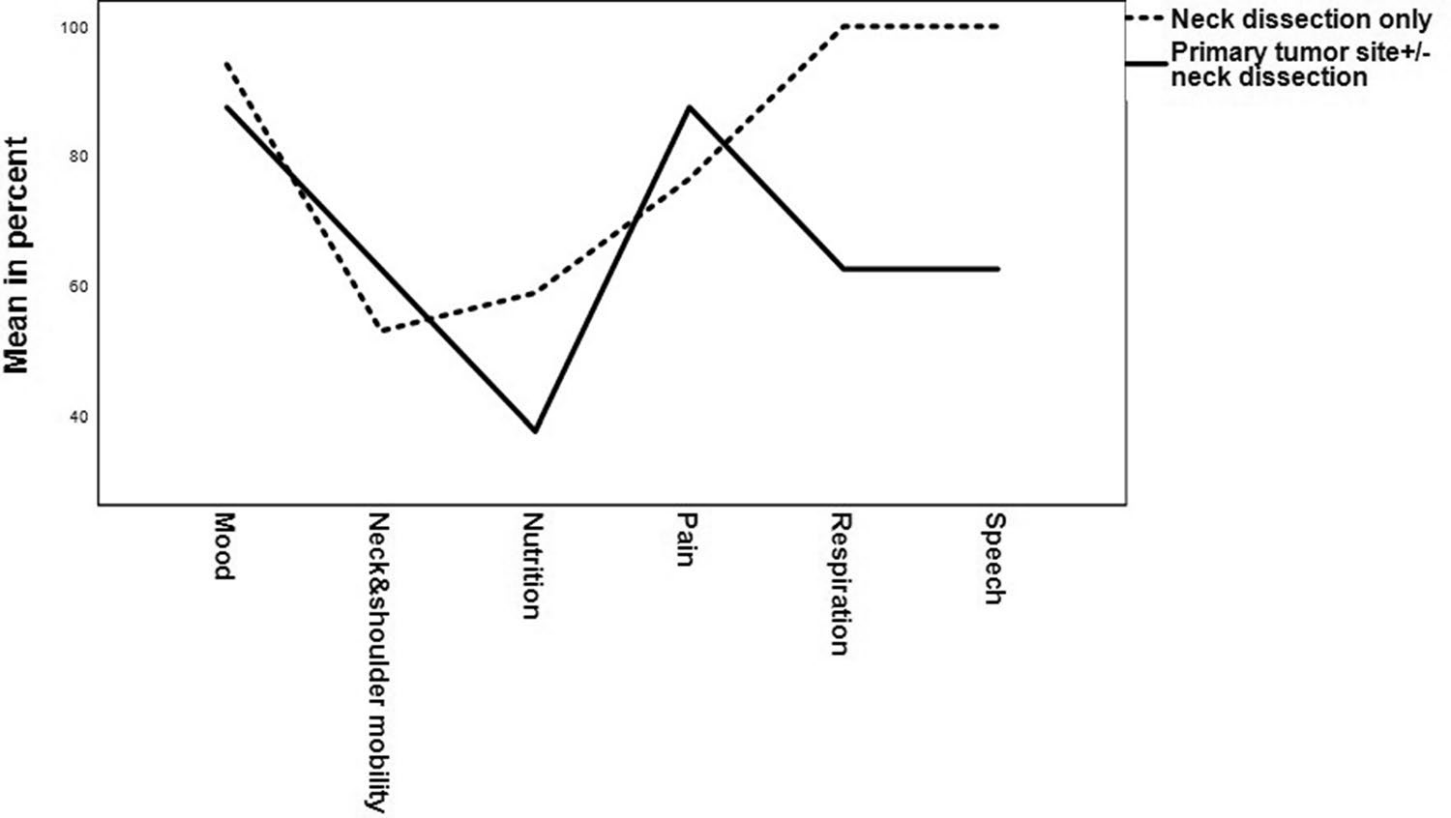
Profile of Head and Neck Cancer Functional Integrity Scale (HNC-FIT Scale). Outcome of Head & Neck Functional Integrity Scale of 25 patients during the last oncological visit following rescue surgery. Rescue surgery was categorized into rescue neck dissection only (dotted line) and resection of the primary tumor with/without neck dissection (solid line). *Y*-axis: percent of patients with complete or almost normal complete functional integrity. *X*-axis: functional domains of the head & neck functional integrity scale

From the 52 patients, 25 were assessed with HNC-Fit scale questionnaire at regular follow-ups. The table in Supplement II informs how many patients have indicated which level of functional integrity. When functional integrity levels 0–2 are combined to severe impairment and grades 3 and 4 to minor or no restriction of functional integrity, a more concise and easier-to-interpret representation of the functional results is obtained. Overall, severe functional impairment was most frequently observed in the functional domains neck and shoulder mobility and nutrition (Fig. 3). Comparing patient with ND only and patients with tumor site surgery ± ND, severe functional impairment occurred significantly more frequent in the functional domains respiration and speech in the latter group (Table 5). Functional outcome was better in patients with initial laryngeal cancer than in patients with any other initial tumor site (*p* = 0.1), especially regarding the domain nutrition (*p* = 0.08).

**Table 5:** Head and neck cancer functional integrity scale items following rescue surgery of persistent HNC in 25 patients

Functional domain	Integrity grade	ND only	Primary site ± ND	Total
Nutrition	Severely impaired	7 (41%)	5 (62%)	12 (48%)
	Normal/slightly impaired	10 (59%)	3 (37%)	13 (52%)
Respiration*	Severely impaired	0 (0%)	3 (37%)	3 (12%)
	Normal/slightly impaired	17 (100%)	5 (63%)	22 (88%)
Speech*	Severely impaired	0 (0%)	3 (37%)	3 (12%)
	Normal/slightly impaired	17 (100%)	5 (63%)	22 (88%)
Pain	Severely impaired	4 (23%)	1 (12%)	5 (20%)
	Normal/slightly impaired	13 (76%)	7 (87%)	20 (80%)
Mood	Severe impaired	1 (6%)	1 (125)	2 (8%)
	Normal/slightly impaired	16 (94%)	7 (87%)	23 (92%)
Neck and shoulder mobility	Severely impaired	8 (47%)	3 (37%)	11 (44%)
	Normal/slightly impaired	9 (53%)	5 (62%)	14 (56%)

^*^Significant differences (*p* < 0.05) in functional integrity between patients with ND only and primary site surgery ± ND

## Discussion

Following first-line treatment of HNC, residual tumor may persist in approximately one fourth of the patients [1, 3, 4]. Second-line surgery, also referred to as rescue or salvage surgery, offers a chance of cure despite first-line treatment failure in patients with resectable disease. We recently reported that patients who received rescue surgery had a better overall survival than patients receiving any other type of second-line therapy [3]. However, rescue surgery is associated with significant morbidity and mortality [1, 5, 19–22]. Clinical data on complications of rescue surgery as well as functional long-term outcome is limited and often restricted to selected surgical procedures like rescue laryngectomies [20, 23, 24]. Moreover, rescue surgery in persistent disease and in recurrent disease is frequently not differentiated [19].

### Study population

We retrospectively studied patients with persistent disease after first-line therapy, who received rescue surgery. Patients with recurrent disease following a previous complete response (CR) were excluded resulting in a low number of 52 included patients. Rescue operations may be performed more easily in patients with persistent HNC shortly after first line treatment, than in patients with recurrent HNC, because postradiogenic tissue fibrosis is less advanced [3]. Of the 52 enrolled patients, 50 had received radiotherapy shortly before as part of their initial treatment and re-irradiation was considered not possible (Table 1). Rescue surgery was separately evaluated for patients who received ND only (*n* = 35) and for patients who received primary site surgery ± ND (*n* = 17). Rescue surgery at the primary tumor site ± ND was a less frequent event, as patients with primary site treatment failure often presented with initial unfavourable disease constellation, which did not allow extensive rescue surgery [20].

Total surgical procedure times differed widely. Overall, the median duration of the surgical rescue procedures was approx. three hours. The median duration of ND only was significantly shorter (less than 3 h) than in primary site surgery ± ND, where surgery lasted approx. 5 h (*p* < 0.001; Table 3). In patients with primary site surgery ± ND without tissue transfer, median duration of the surgical rescue procedures was nearly 3 h and it was significantly longer if reconstruction with tissue transfer was performed (approx. 10 h; *p* < 0.001). Interestingly, procedure times for rescue surgery are in line with those for upfront surgery. For primary neck dissections a surgical procedure time of approx. 3 h has been reported and for primary laryngectomies a median surgical procedure time of approx. 5 h was reported [25–28].

The median length of the hospital stay was 11 days. Patients who received uni- or bilateral neck dissection stayed 9 days, patients who received additionally rescue surgery at the primary tumor site stayed 40 days (*p* < 0.0001). The duration of the hospital stay correlated with the duration of the surgical procedure (*p* < 0.001), requirement of flap reconstruction (*p* = 0.05), a high initial UICC stage (*p* = 0.05) and with the occurrence of postoperative complications (*p* < 0.001). However, inpatient treatment durations vary widely in different countries. Due to structural differences in the health care system and economic pressure, total length of hospital stay recommended for the different surgical procedures are usually lower in the USA than in European countries [29, 30]. In some centres in the USA patients are even discharged home on the first postoperative day after neck dissection, leaving the hospital with suctions drains [31] and a median stay of 8 days was reported for primary laryngectomies [32].

More than one-third of the patients receiving rescue surgery received ICU treatment postoperatively (20/52). Limited transoral or transnasal rescue surgery was routinely performed without postoperative ICU treatment. Postoperative ICU treatment was administered in 20% of patients with second-line ND only, meaning that also for this relatively safe procedure, ICU must be available. Patients with primary site surgery ± ND spent routinely 1 day after surgery on an ICU, especially if free tissue transfer was performed (*p* < 0.001, Table 3). In patients with rescue surgery at the primary tumor site combined with free tissue transfer, a postoperative ICU treatment was three times more likely than in patients with rescue neck dissection only (OR 3.1 ± 0.46, *p* = 0.01). The requirement of ICU after free tissue transfer is discussed controversial. A deep sedation of the patient is often preferred in the initial postoperative period to avoid possible mechanical strain to the transplanted tissues caused by spontaneous movements [33, 34], but also increases the risk of hypoperfusion and flap necrosis because of the decreased systemic blood pressure [33]. In this study all patients with flap reconstruction were observed in an ICU in the postoperative period for safety reasons. As all these patients received a tracheostomy, a mild sedation was possible. The reported average length of stay of ICU after head and neck reconstructive surgery varies considerably between different institutions and countries and up to 11 days have been reported [35–40], but at least 1 day of postoperative ICU treatment is usually recommended [35, 40].

### Mortality and complications associated with rescue surgery

In this population, no perioperative mortality was observed after rescue surgery. In a meta-analysis of Elbers and colleagues from 2019, treatment related mortality was 1% for rescue surgery in HNC [19].

Complications after rescue surgery occurred frequently (21/52, 40%). Severity of complications was classified with the Clavien–Dindo Score (Table 4), a simple and effective system for grading surgical complications in various surgical disciplines including head and neck surgery [18]. If more than one complication occurred, only the complication with the highest Clavien–Dindo Grade was counted, as this was usually the complication with the greatest impact on the postoperative course and patients were often developing complications of increasing severity depending one from each other [11] like, e.g. impaired wound healing (Clavien Dindo grade II) that caused pharyngocutaneus fistula formation with the necessity of surgical intervention (grade III). Most patients suffering from complications were affected by more than one complication (16/21; 76%). Grade III complications requiring invasive intervention were observed in nearly every fifth patient and life-threatening complications grad IV were encountered in nearly 10% of the patients (Table 4). The high risk of surgical complications is the decisive disadvantage of rescue surgery, making it a double-edged sword [20].

According to the meta-analysis performed by Goodwin and colleagues the complication rate of rescue surgery at all tumor sites ranges up to 39% [20]. In this meta-analysis, studies are included that date back to the early 1970s. As surgical techniques have improved, especially the availability of myocutaneous flaps as well as free flaps with microvascular anastomosis, surgeons have the opportunity to bring unirradiated tissue into the surgical filed for reconstruction, facilitating wound healing. However flap reconstruction caries it own risks [41] and patients that received flap reconstruction were more likely to have postoperative complications (*p* = 0.01).

Rescue laryngectomies are related with a lower complication rate, than rescue surgeries at other tumor sites, were complication rates range up to 62% [5, 21, 22]. In line with the literature, in our study cohort patients with initial laryngeal cancer were less likely to suffer from postoperative complications than patients with any other primary tumor sites (*p* = 0.1).

Negative predictors for postoperative complications were higher ASA score, UICC stages and the prior received radiotherapy dose in Gray. Patients with ND only were less likely to have postoperative complications than patients with primary site surgery ± ND (*p* < 0.01). Rescue neck dissection is in general regarded as safe and effective procedure but even there complications rates are described as high as 28% in irradiated patients, comparable with the 25% of patients with ND only who had postoperative complications in this study cohort [24, 42].

### Overall survival after rescue surgery

Median overall survival of patients receiving rescue surgery was 56 months (95% CI 44–69 months). In a previous study, we reported that patients with persistent disease who received rescue surgery had a significantly better median overall survival in comparison with patients receiving any other type of second-line treatment, like systemic therapy or second-line radiotherapy [3]. Information about overall survival after rescue surgery in HNC in general are sparse and were especially reported after selected surgical procedures, like rescue laryngectomies. After rescue laryngectomy an overall survival of 32 months was reported [43] and after rescue neck dissection in patients with only regional disease a 5-year survival of 55% was described [24]. Patients with cancer of the oral cavity (18 months) or the larynx (31 months) were described to have the best median overall survival after rescue surgery when compared to patients with any other primary tumor site [20, 44, 45].

In this study cohort median overall survival was also best for patients with laryngeal cancer (72 months) and patients with oropharyngeal cancer (63 months). These better outcome results in comparison with the literature might be biased by the fact that only patients with tumor persistence, but not with tumor recurrence, were included in the study. Tumor persistence is evaluated in our department 8–10 weeks after first-line therapy allowing early detection and initiation of rescue surgery. Tumor relapse is often asymptomatic and the time of detection is dependent on the frequency of oncological follow-up intervals [1].

Furthermore, the better overall survival of patients with initial laryngeal cancer is biased by the fact that patients with low UICC stages of laryngeal carcinoma that were treated with small field irradiation as first-line treatment are also included in this study (*n* = 3). The rescue treatment consisted of transoral laser microsurgery. These patients have-due to their initial favourable disease constellation—a longer overall survival.

Median overall survival after rescue surgery was significantly better for patients with ASA score I/II than for patients with an ASA score III/IV (*p* = 0.04). A further positive predictor for overall survival after rescue surgery was a postoperative course without complications (*p* = 0.05, Fig. 2). The influence of complications on overall survival after rescue surgery could be biased by the fact that especially patients with higher initial UICC stages were more likely to have postoperative complications.

### Functional outcome after rescue surgery

The HNC-FIT scale is used to record the functional integrity of patients with HNC (suppl. 1 and 2). The HNC-Fit scale has six functional domains and five ordinal descriptors for functional outcome. The descriptors do not capture the subjective estimate of quality of life, but objective external indicators of functional integrity. Each functional domain is individually recorded and evaluated; no sum scores are formed (suppl. 1 and 2). Rather, the evaluation is intentionally limited to the basic representation of the absolute or relative number of patients, for whom the respective functional descriptor applies. By representing the percentage of patients who have achieved complete or almost complete functional integrity in the respective functional dimension, one obtains a profile plot of the functional outcome (Fig. 3). In the evaluated patients, 12/25 had severely impaired nutritional function, 11/25 had severely impaired neck and shoulder mobility and 5/25 had severe pain.

There are several publications that reported that rescue surgery in general noticeably reduced functional integrity. Especially normalcy of diet, understandably of speech and public eating behaviour were severely affected by rescue surgery [20]. Similarly in this study functional deficits were mainly observed in the functional domains speech, neck, shoulder and arm mobility and nutrition. The least impairment was observed in the functional domains mood, pain and respiration (Fig. 3). Rescue neck dissection in general was a functional well-tolerated and safe procedure. Patients who received a rescue neck dissection were two times less likely to have functional impairment than patients who received a rescue surgery at the primary tumor site (OR 2.5 ± 2; *p* = 0.07). The primary tumor site correlated with the functional impairment, as patients with initial laryngeal cancer had the best functional outcome after rescue surgery when compared with any other tumor site (*p* = 0.1), especially regarding the domain nutrition (*p* = 0.08). Accordingly, rescue laryngectomy was described as a functional well-tolerated surgery especially regarding swallowing function [20, 45]. Furthermore, the functional integrity of these patients correlated significantly with the initial UICC stage (*p* = 0.05), the received RT dose during first-line treatment (*p* = 0.05) but it did not correlated with arising complications associated with rescue surgery (*p* = 0.6).

## Conclusion

Rescue surgery offers selected patients with persistent disease after first-line therapy a realistic chance to achieve complete response and long-term survival. Especially, patients with persistent laryngeal and oropharyngeal cancer profit from rescue surgery. Rescue neck dissection is a safe, effective and functionally well-tolerated procedure in patients with regional persistence. However, rescue surgery at the primary tumor site ± neck dissection carries a high morbidity resulting in long hospital stays and patients should expect long-term functional impairment.

## Electronic supplementary material

Below is the link to the electronic supplementary material.

Supplementary file1 (PDF 64 kb)

Supplementary file2 (PDF 58 kb)

Supplementary file3 (PDF 62 kb)

## References

[1] Pagh A, Grau C, Overgaard J (2016). Failure pattern and salvage treatment after radical treatment of head and neck cancer. Acta Oncol.

[2] Adelstein D, Gillison ML, Pfister DG, Spencer S, Adkins D, Brizel DM (2017). NCCN Guidelines insights: head and neck cancers, version 2.2017. J Natl Compr Cancer Netw.

[3] Steinbichler TB, Lichtenecker M, Anegg M, Dejaco D, Kofler B, Schartinger VH (2018). Persistent head and neck cancer following first-line treatment. Cancers.

[4] Yovino S, Settle K, Taylor R, Wolf J, Kwok Y, Cullen K (2010). Patterns of failure among patients with squamous cell carcinoma of the head and neck who obtain a complete response to chemoradiotherapy. Head Neck.

[5] Esteller E, Vega MC, Lopez M, Quer M, Leon X (2011). Salvage surgery after locoregional failure in head and neck carcinoma patients treated with chemoradiotherapy. Eur Arch Otorhinolaryngol.

[6] NCCN Guidelines Version 2.2018 Head and Neck Cancers. Clinical practice guidelines in oncology (NCCN guidelines). 2018 06.09.2018; 2.2018(06/20/18)

[7] Lango MN, Myers JN, Garden AS (2009). Controversies in surgical management of the node-positive neck after chemoradiation. Semin Radiat Oncol.

[8] Schouten CS, Hoekstra OS, Leemans CR, Castelijns JA, de Bree R (2015). Response evaluation after chemoradiotherapy for advanced staged oropharyngeal squamous cell carcinoma: a nationwide survey in the Netherlands. Eur Arch Otorhinolaryngol.

[9] Deschler DG MM, Smith RV (eds) (2014) TNM staging of head and neck cancer and neck dissection classification, 4th edition: American Academy of Otolaryngology-Head and Neck Surgery Foundation

[10] Dindo D, Demartines N, Clavien PA (2004). Classification of surgical complications: a new proposal with evaluation in a cohort of 6336 patients and results of a survey. Ann Surg.

[11] Clavien PA, Barkun J, de Oliveira ML, Vauthey JN, Dindo D, Schulick RD (2009). The Clavien–Dindo classification of surgical complications: 5-year experience. Ann Surg.

[12] Mak PH, Campbell RC, Irwin MG, American Society of A (2002) The ASA physical status classification: inter-observer consistency American Society of Anesthesiologists. Anaesth Intensiv Care 30(5):633–40 10.1177/0310057X0203000516 12413266

[13] TNM classification of malignant tumours. Leslie H. Sobin MKG, Christian Wittekind, editor: UICC; 2011

[14] Kofler B, Borena W, Manzl C, Dudas J, Wegscheider AS, Jansen-Durr P (2017). Sensitivity of tumor surface brushings to detect human papilloma virus DNA in head and neck cancer. Oral Oncol.

[15] Reimers N, Kasper HU, Weissenborn SJ, Stutzer H, Preuss SF, Hoffmann TK (2007). Combined analysis of HPV-DNA, p16 and EGFR expression to predict prognosis in oropharyngeal cancer. Int J Cancer.

[16] Schemper M, Smith TL (1996). A note on quantifying follow-up in studies of failure time. Control Clin Trials.

[17] Burgette LF, Mulcahy AW, Mehrotra A, Ruder T, Wynn BO (2017). Estimating surgical procedure times using anesthesia billing data and operating room records. Health Serv Res.

[18] Monteiro E, Sklar MC, Eskander A, de Almeida JR, Shrime M, Gullane P (2014). Assessment of the Clavien–Dindo classification system for complications in head and neck surgery. Laryngoscope.

[19] Elbers JBW, Veldhuis LI, Bhairosing PA, Smeele LE, Jozwiak K, van den Brekel MWM et al (2019) Salvage surgery for advanced stage head and neck squamous cell carcinoma following radiotherapy or chemoradiation. Eur Arch Oto-Rhino-Laryngol 10.1007/s00405-019-05292-0 30673847

[20] Goodwin WJ (2000) Salvage surgery for patients with recurrent squamous cell carcinoma of the upper aerodigestive tract: when do the ends justify the means? Laryngoscope 110(3 Pt 2 Suppl 93):1–18 10.1097/00005537-200003001-00001 10714711

[21] Leon X, Aguero A, Lopez M, Garcia J, Farre N, Lopez-Pousa A (2015). Salvage surgery after local recurrence in patients with head and neck carcinoma treated with chemoradiotherapy or bioradiotherapy. Auris Nasus Larynx.

[22] Tan HK, Giger R, Auperin A, Bourhis J, Janot F, Temam S (2010). Salvage surgery after concomitant chemoradiation in head and neck squamous cell carcinomas—stratification for postsalvage survival. Head Neck.

[23] Hilly O, Gil Z, Goldhaber D, Amit M, Biadsee A, Popovtzer A (2015). Elective neck dissection during salvage total laryngectomy—a beneficial prognostic effect in locally advanced recurrent tumours. Clin Otolaryngol.

[24] van den Bovenkamp K, Noordhuis MG, Oosting SF, van der Laan B, Roodenburg JL, Bijl HP (2017). Clinical outcome of salvage neck dissections in head and neck cancer in relation to initial treatment, extent of surgery and patient factors. Clin Otolaryngol.

[25] Duran-Briones G, Gallegos Hernandez JF, Rendon Arroyo ME, Hernandez-Hernandez DM (2011). Drainage time in patients submitted to radical neck dissection Influence of peri-operative intravenous liquid perfusion. Gac Med Mex.

[26] Verma RK, Mathiazhagan A, Panda NK (2017). Neck dissection with harmonic scalpel and electrocautery? A randomised study. Auris Nasus Larynx.

[27] Zhang D, Xie L, He G, Fang L, Miao Y, Wang Z (2017). A comparative study of the surgical outcomes between video-assisted and open lateral neck dissection for papillary thyroid carcinoma with lateral neck lymph node metastases. Am J Otolaryngol..

[28] E R. Laryngectomy: purpose, procedure, and recovery. Healthline. 2017.

[29] Fuchs VR (2013). How and why US health care differs from that in other OECD countries. JAMA.

[30] Tedesco D, Hernandez-Boussard T, Carretta E, Rucci P, Rolli M, Di Denia P (2016). Evaluating patient safety indicators in orthopedic surgery between Italy and the USA. Int J Qual Health Care.

[31] Ha PK, Couch ME, Tufano RP, Koch WM, Califano JA (2005). Short hospital stay after neck dissection. Otolaryngol Head Neck Surg.

[32] Goepfert RP, Hutcheson KA, Lewin JS, Desai NG, Zafereo ME, Hessel AC (2017). Complications, hospital length of stay, and readmission after total laryngectomy. Cancer.

[33] Nkenke E, Vairaktaris E, Stelzle F, Neukam FW, St PM (2009). No reduction in complication rate by stay in the intensive care unit for patients undergoing surgery for head and neck cancer and microvascular reconstruction. Head Neck.

[34] Nunes S, Berg L, Raittinen LP, Ahonen H, Laranne J, Lindgren L (2007). Deep sedation with dexmedetomidine in a porcine model does not compromise the viability of free microvascular flap as depicted by microdialysis and tissue oxygen tension. Anesth Analg.

[35] de Melo GM, Ribeiro KC, Kowalski LP, Deheinzelin D (2001). Risk factors for postoperative complications in oral cancer and their prognostic implications. Arch Otolaryngol Head Neck Surg.

[36] Klug C, Berzaczy D, Reinbacher H, Voracek M, Rath T, Millesi W (2006). Influence of previous radiotherapy on free tissue transfer in the head and neck region: evaluation of 455 cases. Laryngoscope..

[37] McCrory AL, Magnuson JS (2002). Free tissue transfer versus pedicled flap in head and neck reconstruction. Laryngoscope..

[38] O'Neill JP, Shine N, Eadie PA, Beausang E, Timon C (2010). Free tissue transfer versus pedicled flap reconstruction of head and neck malignancy defects. Ir J Med Sci.

[39] Pohlenz P, Blessmann M, Blake F, Li L, Schmelzle R, Heiland M (2007). Outcome and complications of 540 microvascular free flaps: the Hamburg experience. Clin Oral Investig.

[40] Ryan MW, Hochman M (2000). Length of stay after free flap reconstruction of the head and neck. Laryngoscope..

[41] Lahtinen S, Koivunen P, Ala-Kokko T, Kaarela O, Ohtonen P, Laurila P (2018). Complications and outcome after free flap surgery for cancer of the head and neck. Br J Oral Maxillofac Surg.

[42] Chung EJ, Lee SH, Baek SH, Bae WJ, Chang YJ, Rho YS (2015). Clinical outcome and prognostic factors after salvage surgery for isolated regional squamous cell carcinoma recurrences. Head Neck.

[43] Fletcher KT, Gal TJ, Ebelhar AJ, Valentino J, Brill YM, Dressler EV (2017). Prognostic indicators and survival in salvage surgery for laryngeal cancer. Head Neck.

[44] Chung EJ, Park MW, Kwon KH, Rho YS (2019) Clinical outcomes and prognostic factor analysis after salvage surgery for recurrent squamous cell carcinoma of the oral cavity. Int J Oral Maxillofac Surg 10.1016/j.ijom.2019.03.967 31492478

[45] Mimica X, Hanson M, Patel SG, McGill M, McBride S, Lee N (2019). Salvage surgery for recurrent larynx cancer. Head Neck.

