# Supplementary material for: Surgical rescue for persistent head and neck cancer after first-line treatment
Source: Eur Arch Otorhinolaryngol. 2020 Jan 25;277(5):1437–48. doi: 10.1007/s00405-020-05807-0 (PMC7160075; doi:10.1007/s00405-020-05807-0)
Supplement: Supplementary file 1 — Supplementary file1 (PDF 64 kb) [file 405_2020_5807_MOESM1_ESM.pdf]

Suppl. 1: Head and Neck Cancer Functional Integrity Scale (HNC-FIT Scale)

The Head and Neck Cancer Functional Integrity Scale (HNC-FIT Scale) is completed by the physician during the patient interview at regular follow-up visits and serves for internal quality control. It needs normally less than 2 minutes to fill in the questionnaire form. The HNC-FIT Scale covers the functional domains nutrition, respiration, speech, pain, mood, and neck and shoulder mobility. Functional integrity is scaled from 0 to 4, with 0 meaning complete loss of normal function and 4 meaning functional integrity as it was before the disease began.

**Functional domain**	**Integrity Grade**				
	**0**	**1**	**2**	**3**	**4**
**Nutrition**	Unable to swallow; only via gastrostomy tube	Gastrostomy tube needed, some oral feeding possible	No gastrostomy tube, oral diet, but only liquid/soft food	No gastrostomy tube, diet slightly restricted	normal
**Respiration**	Tracheostoma, needs blocked cannula	Tracheostoma, speech cannula/no cannula	No tracheostoma, breathing difficulties at rest	No tracheostoma, breathing difficulties only on exertion	normal
**Speech**	Not possible, without phonation	Difficult to understand, no phone calls	Telephoning possible	Easy to understand, but pronunciation/voice changed	normal
**Pain**	Pain despite opiate therapy	Controlled with opiates	Regularly needs non-opioid analgesics	Needs analgesics from time to time	normal
**Mood**	Suicidal thoughts	Very depressed despite antidepressants	With antidepressants overall normal mood	Occasionally depressed, no antidepressants needed	normal
**Neck & shoulder mobility**[1)]	Stiff neck and/or shoulder, hardly any movement possible	Can hair hardly comb, looking backwards in car not possible	Combing with problems, looking backwards in car difficult	Combing and looking backwards in car slightly restricted	normal

1) The worse result of neck mobility and shoulder mobility is counted
