# Supplementary material for: Surgical rescue for persistent head and neck cancer after first-line treatment
Source: Eur Arch Otorhinolaryngol. 2020 Jan 25;277(5):1437–48. doi: 10.1007/s00405-020-05807-0 (PMC7160075; doi:10.1007/s00405-020-05807-0)
Supplement: Supplementary file 2 — Supplementary file2 (PDF 58 kb) [file 405_2020_5807_MOESM2_ESM.pdf]

Suppl. 2: Absolute and relative number of patients and grade of functional integrity in the 6 functional domains Nutrition, Respiration, Speech, Pain, Mood, and Neck&shoulder mobility.

		Count	Column N %
Nutrition	Unable to swallow; only via gastrostomy tube	3	12%
	Via gastrostomy tube and oral	7	28%
	No gastrostomy tube, oral but only liquid/soft food	2	8%
	No gastrostomy tube, diet slightly restricted	6	24%
	normal	7	28%
Respiration	Tracheostoma, blocked cannula	0	0%
	Tracheostoma, speech cannula/no cannula	3	12%
	No tracheostoma, breathing difficulties at rest	0	0%
	No tracheostoma, breathing difficulties only on exertion	7	28%
	normal	15	60%
Speech	Not possible, without phonation	2	8%
	difficult to understand, no phone calls	1	4%
	Telephoning possible	0	0%
	Easy to understand, but pronunciation/voice changed	14	56%
	normal	8	32%
Pain	Pain despite opiate therapy	0	0%
	Controlled with opiates	3	12%
	Regularly needs non-opioid analgesics	2	8%
	Needs analgesics from time to time	5	20%
	no pain	15	60%
Mood	Suicidal thoughts	0	0%
	Very depressed despite antidepressants	0	0%
	With antidepressants overall normal mood	2	8%
	Occasionally depressed mood, no antidepressants needed	5	20%
	normal	18	72%
Neck&shoulder mobility	Stiff neck, hardly any movement possible	0	0%
	Can hair hardly comb and/or looking backwards in car not possible	3	12%
	Combing with problems and/or looking backwards in car difficult	8	32%
	Combing and/or looking backwards in car slightly restricted	8	32%
	normal	6	24%
