# Supplementary material for: Surgical rescue for persistent head and neck cancer after first-line treatment
Source: Eur Arch Otorhinolaryngol. 2020 Jan 25;277(5):1437–48. doi: 10.1007/s00405-020-05807-0 (PMC7160075; doi:10.1007/s00405-020-05807-0)
Supplement: Supplementary file 3 — Supplementary file3 (PDF 62 kb) [file 405_2020_5807_MOESM3_ESM.pdf]

Suppl. 3: Instructions for physicians of the Department of Otorhinolaryngology – Head and Neck Surgery, Medical University of Innsbruck for completing the Head and Neck Functional Integrity Scale

In the functional domain nutrition, 'Unable to swallow; only via gastrostomy tube' means that the patient is unable to swallow, and his intake of food is exclusively via gastrostomy tube. This applies also for nasogastric tubes. 'Gastrostomy tube needed, some oral feeding possible' means that the patient relies on PEG for adequate food and fluid intake, but occasional sips are possible. 'No gastrostomy tube, oral diet, but only liquid/soft food' means that the patient does not have a gastrostomy tube but cannot eat solid food. 'No gastrostomy tube, diet slightly restricted' means that the patient can essentially eat normally, but with certain (e.g. dry foods) problems, but these can be overcome (for example, by simultaneously taking liquid). 'Normal', like in all functional domains, means that the function is as it was before the illness.

In the functional domain respiration 'Tracheostoma, needs blocked cannula' means that the patient has a tracheostoma and must use a cannula with blocked cuff, e.g. because of aspiration. 'Tracheostoma, speech cannula/no cannula' means a simple condition with tracheostomy. 'No tracheostoma, breathing difficult at rest' is ticked at dyspnoea at rest. With 'No tracheostoma, breathing difficulties only on exertion', a typical loading situation would be e.g. climb stairs.

In the functional domain speech 'Not possible, without phonation' means that the patient is incapable of speech and essentially must rely on written communication. At 'Difficult to understand, no phone calls', the patient can indeed make sounds and you can understand the meaning with high concentration, but the patient in not able to make calls. At 'Telephoning possible' is the language is very limited and difficult to understand, but it is a communication over the phone with strangers possible. 'Easy to understand, but pronunciation/voice changed' also detects slight functional limitations of speech, but the communication is possible without any problems.

In the pain dimension 'pain despite of opiate therapy" means that the patient suffers from pain despite pain therapy with opiates by experienced pain specialists. 'Controlled with opiates' means that with adequate pain therapy including opiates the patient is essentially painless. 'Regularly needs non-opioid analgesics' means that the patient has a long-term pain therapy without opioids and is thus essentially painless. 'Needs analgesics from time to time' means occasional pain and occasionally use of painkillers.

In the dimension mood, 'suicidal thoughts' means that the patient is so depressed that he has suicidal thoughts. As a rule, psychiatric intervention is urgently needed. 'Very depressed despite antidepressants' means that the patient has sustained depressive mood despite adequate antidepressant therapy received by a suitable medical facility. 'With antidepressants overall normal mood' means that the patient regularly needs antidepressants because of depressive mood. With this antidepressant therapy, however, the mood is essentially normal. 'Occasionally depressed, no antidepressants needed' is ticked in case of occasional depressive mood.

In the shoulder-neck-mobility dimension two functions are queried simultaneously, namely shoulder mobility and neck mobility. In each case the worse functional status is ticked. 'Stiff neck and/or shoulder, hardly any movement possible" is a complete fixation in the neck and/or shoulder area. 'Can hair hardly comb, looking

backwards in car not possible' means that the shoulder mobility is so severely limited that with a straight head not all areas of the head are reachable with the comb and/or that without a rear view camera the car cannot be reversed under visual control because the head cannot be turned far enough. 'Combing with problems, looking backwards in car difficult' means that combing the hair when the head is straight and that reversing under sight is possible, but means a considerable effort. 'Combing and looking backwards in car slightly restricted' means a slight restriction of the neck and shoulder mobility without significant functional impairment.
